# Prevalence and Risk Factors for *Pearsonema plica* Infection in Hunting Dogs in Serbia

**DOI:** 10.3390/ani15203025

**Published:** 2025-10-18

**Authors:** Tamara Ilic, Nemanja M. Jovanovic, Tamas Petrovic, Predrag Stepanovic, Darko Despotovic, Katarina Nenadovic, Vladimir Gajdov, Natalija Fratric, Jelena Aleksic Radojkovic

**Affiliations:** 1 Department of Parasitology, Faculty of Veterinary Medicine, University of Belgrade, Bul. Oslobodjenja 18, 11000 Belgrade, Serbia; tamara@vet.bg.ac.rs; 2 Scientific Veterinary Institute “Novi Sad”, Rumenacki put 20, 21113 Novi Sad, Serbia; tomy@niv.ns.ac.rs (T.P.); vladimir.g@niv.ns.ac.rs (V.G.); 3 Department of Equine, Small Animal, Poultry and Wild Animal Diseases, Faculty of Veterinary Medicine, University of Belgrade, Bul. Oslobodjenja 18, 11000 Belgrade, Serbia; pedja@vet.bg.ac.rs; 4 PI Veterinary Institute of the Republic of Srpska “Dr. Vaso Butozan”, 78000 Banja Luka, Bosnia and Herzegovina; darkodespotovicbl@gmail.com; 5 Department of Animal Hygiene, Faculty of Veterinary Medicine, University of Belgrade, Bul. Oslobodjenja 18, 11000 Belgrade, Serbia; katarinar@vet.bg.ac.rs; 6 Department of Physiology and Biochemistry, Faculty of Veterinary Medicine, University of Belgrade, Bul. Oslobodjenja 18, 11000 Belgrade, Serbia; nataly@vet.bg.ac.rs; 7 Department of Forensic Veterinary Medicine, Faculty of Veterinary Medicine, University of Belgrade, Bul. Oslobodjenja 18, 11000 Belgrade, Serbia; alexjellena@vet.bg.ac.rs

**Keywords:** *Pearsonema plica*, hunting dogs, prevalence, risk factors, molecular characterization

## Abstract

**Simple Summary:**

Urinary capillariosis, caused by the nematode *Pearsonema plica*, affects the urinary tracts of dogs but often goes undetected due to mild or absent symptoms. This study examined hunting dogs in Serbia to determine the prevalence and risk factors of infection. Eggs of *P. plica* were found in 20.45% of the animals, and molecular analysis confirmed the parasite. Hunting activity and irregular deworming were identified as key risks. Recognizing this parasite is important for timely veterinary diagnosis and prevention.

**Abstract:**

*Pearsonema plica* is a nematode commonly found in wild carnivores and occasionally in domestic dogs, where infections are often overlooked. This cross-sectional study investigated its prevalence in 88 hunting dogs from five districts in Serbia between October 2021 and May 2024. Urine samples were examined via light microscopy, and molecular analyses (PCR and sequencing of the 18S rRNA gene). Presence of *P. plica* eggs was found in 20.45% of the tested dogs. Phylogenetic analysis clustered the obtained isolates with reference sequences of *P. plica*. Hunting activity within two months prior to sampling and irregular or infrequent deworming were significantly associated with higher infection rates. Dogs showing urinary symptoms were more likely to test positive. This study provides the first molecularly confirmed data on *P. plica* infection in hunting dogs in Serbia and indicates that urinary capillariosis in dogs and the need for greater clinical awareness. However, due to the limited sample size and potential sampling bias, the results should be interpreted with caution. Further large-scale and longitudinal studies are needed to better understand the epidemiology and clinical relevance of this infection in domestic dogs.

## 1. Introduction

The nematode *Pearsonema* (syn. *Capillaria*) *plica* is a common parasite found in the urinary tracts of both domestic and wild carnivores. Infections frequently occur without significant clinical symptoms, which can lead to misunderstandings in diagnosis among veterinarians. However, when the infection is complicated by secondary bacterial infections, noticeable clinical signs may emerge. The primary reservoir of the infection for domestic carnivores is the red fox. This species, along with infected intermediate hosts such as terrestrial oligochaetes (phylum Annelida) and paratenic hosts like rodents and birds, poses a potential risk of infection for hunting dogs and household pets [1].

*Pearsonema plica* is commonly found among carnivores worldwide. In Europe, it has been most frequently reported in red foxes (*Vulpes vulpes*) [2,3,4]. It is less commonly seen in wolves (*Canis lupus*) [5], brown bears (*Ursus arctos*) [6], golden jackals (*Canis aureus*) [1], some species of martens (*Martes* spp.) [7], and raccoons (*Procyon lotor*) [8]. The number of reported cases of urinary capillariosis in dogs and cats is relatively low, with existing studies primarily describing isolated cases of infection in dogs diagnosed in the United States [9,10] and Europe [11,12,13,14,15,16,17,18,19,20,21,22].

Infections often have low intensity and are associated with mild or non-specific clinical symptoms, which can lead to the disease going undiagnosed. Although there are few epidemiological studies regarding the prevalence of *P. plica* in European countries, we cannot rule out the possibility of its wider presence and more significant parasitism in dogs and cats in this region [16,20].

Hunting dogs, due to their activities, are generally exposed to numerous pathogens and parasites. Hunting activities represent an important risk factor that can influence patterns of parasite infections in dogs. Transmission risk arises when hunting dogs are taken into habitats inhabited by wild carnivores or when they are fed game meat by their owners [23,24,25]. Also, transmission might either occur directly from wild carnivores via the environment to hunting dogs (no intermediate host required) or indirectly via intermediate hosts. Although numerous studies have been published on parasites in companion dogs [26,27], the parasite fauna of hunting dogs remains largely understudied. In our previous research, we observed that hunting dogs are at higher risk of intestinal endoparasite infections [28]. Therefore, the present study aims to determine the presence and perform molecular characterization of the nematode *P. plica* in hunting dogs in Serbia. A further aim is to evaluate the impact of various risk factors that may contribute to the occurrence, persistence, and spread of urinary capillariosis in this population.

## 2. Materials and Methods

### 2.1. The Study Area

**Figure 1:**
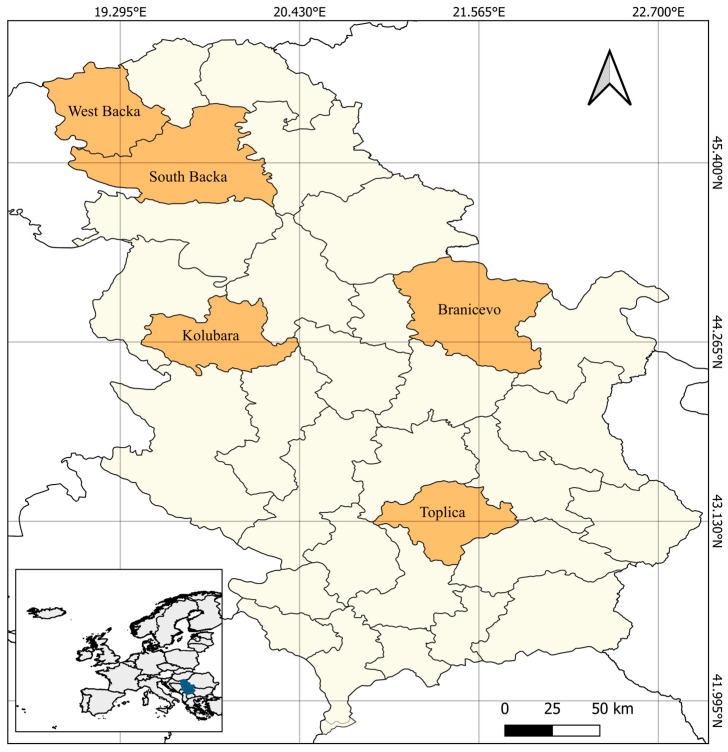
Administrative district where the study was carried out.

A clinical and parasitological examination was conducted on 88 hunting dogs, which were used for locating, capturing, and retrieving game animals. The study was carried out across five administrative districts of the Republic of Serbia: West Backa, South Backa, Branicevo, Kolubara, and Toplica (Figure 1).

### 2.2. Sampling and Examination of Urine

Urine samples from dogs were collected between October 2021 and May 2024 during the spring, autumn, and winter seasons. Dogs were recruited through collaborating veterinary clinics during both routine and problem-oriented visits. Inclusion criteria encompassed hunting dogs that were active in field conditions (exposed to natural habitats of potential intermediate hosts), regardless of ownership or age. Two categories of dogs were examined: (1) animals that exhibited clinical symptoms of urinary tract disorders (such as polydipsia, polyuria, dysuria, hematuria), and (2) dogs that engaged in the same activities and frequented the same environments as the symptomatic animals but did not show any signs of urinary tract disease. This approach aimed to increase the probability of detecting infection with *P. plica* within the exposed hunting dog population.

Urine samples were collected from dogs either by spontaneous urination or by cystocentesis into sterile plastic containers by licensed veterinarians. For spontaneous collection, urine was obtained in a sterile container during a walk. Briefly, the external genitalia were inspected, visible dirt and hair were removed, and the prepuce or vulva was flushed with sterile isotonic saline prior to collecting the free-catch urine sample. Cystocentesis was performed by antepubic puncture of the urinary bladder through the abdominal wall.

Samples were transported to the laboratory of the Department of Parasitology at the Faculty of Veterinary Medicine, University of Belgrade, in portable refrigerators at a temperature of +4 °C. All samples were examined within 24 h. For the parasitological analyses, the urine samples were centrifuged for 5 min at 1500 rpm, after which the supernatant was discarded. The sediment was then examined using a light microscope (Olympus CX23, Tokyo, Japan) at magnifications of 100× and 400×. All detected parasitic elements were photographed.

### 2.3. DNA Extraction, PCR Amplification and Sanger Sequencing

Two urine samples containing urinary nematodes *Pearsonema* eggs were chosen for molecular analysis and species confirmation. For the detection of *Pearsonema* genome presence, the urine samples were centrifugated at 5000× *g* and after carefully removing the supernatants, the sediments were collected and transferred to 2 mL micro tubes and homogenized in 1 mL sterile phosphate-buffered saline, with 0.2 g of quartz sand and one steel bead (4 mm) for 3 min using a TissueLyser LT (Qiagen, Hilden, Germany) operating at 50 Hz. The homogenates were then centrifuged for 10 min at 2000× *g*, and the supernatant was used for DNA extraction. Genomic DNA was extracted using the commercial kit IndiSpin Pathogen Kit (Indical Bioscience GmbH, Leipzig, Germany) according to the manufacturer’s instructions.

A partial fragment of the 18S rRNA gene of ∼620 bp was amplified using PCR with primers specific to nematode 18S regions [29]. The PCR reaction was performed using the HotStarTaq DNA Polymerase kit (Qiagen, Germany), with a small modification of the manufacturer’s instructions. Briefly, the amplification reaction was carried out in a volume of 25 μL containing 3 μL of DNA, 12.5 μL of HotStar Taq Master Mix and 25 pmol of each primer. The amplification conditions were as follows: initial denaturation at 95 °C for 15 min; 40 cycles of 95 °C for 30 s, 53 °C for 30 s, and 72 °C for 1 min; followed by a final extension at 72 °C for 10 min. Amplified products were verified via agarose gel electrophoresis and purified using the GeneJET PCR Purification kit (Thermo Fisher Scientific, Waltham, MA, USA). Sanger sequencing was performed in both directions using the same primers as a commercial service (Macrogen, Europe, Amsterdam, The Netherlands https://www.macrogen-europe.com/).

### 2.4. Sequence Processing and Phylogenetic Analysis

Raw sequence chromatograms were visualized, edited, and assembled into consensus sequences using the Staden package [30]. Low-quality bases and primer sequences were trimmed, and consensus sequences were generated. Phylogenetic reconstruction was carried out using MEGA11 [31]. A phylogenetic tree was generated in MEGA11 [31]. The best-fit nucleotide substitution model identified by MEGA model test was K2 (Kimura 2) with uniform rates among sites. Maximum-likelihood inference was performed in MEGA11 under this model, with 1000 bootstrap replicates. The sequence data generated in this paper have been deposited in NCBI GenBank with the following accession numbers: PV596675 and PV596676.

### 2.5. Risk Factor Assessments

The study investigated the impact of various individual and environmental risk factors. Specifically, it analyzed specific parameters related to sex (male and female) and age groups (<2 years, 2–8 years, >8 years). The evaluation of environmental risk factors included several parameters: type of diet (commercial, mixed, and combined), housing conditions (kept in a yard, in a kennel with an exercise area, or in a standard kennel), hunting activity (last hunting trip occurred 2, 4, or 6 months before sampling), deworming history (regular—2 to 4 times per year, occasional—once per year, and irregular—not conducted for over one year), presence of urinary symptoms (yes or no), method of urine collection (spontaneous urination or cystocentesis), season of sample collection (autumn, winter, and spring), and administrative district (West Backa, South Backa, Branicevo, Kolubara, and Toplica).

### 2.6. Statistical Analyses

The results were analyzed using GraphPad Prism software, version 7 (GraphPad, San Diego, CA, USA). To identify potential risk factors associated with Capillaria infection in dogs, a multinominal logistic regression analysis was performed, which provides exact regression estimates, 95% confidence intervals, odds ratios, and *p*-values. This method allows for the assessment of categorical outcomes with more than two levels and provides adjusted estimates for each predictor while accounting for the effects of other variables. Statistical significance was set at *p* < 0.05.

## 3. Results

**Figure 2:**
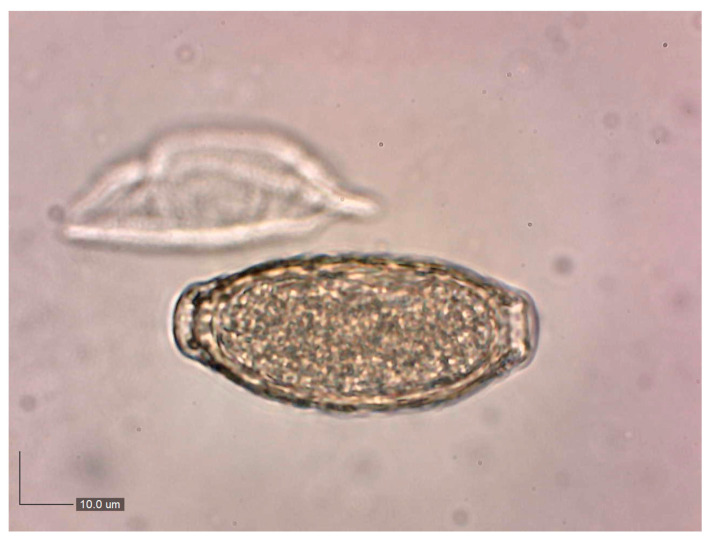
*Pearsonema plica* egg isolated from urine sediment (400×).

**Figure 3:**
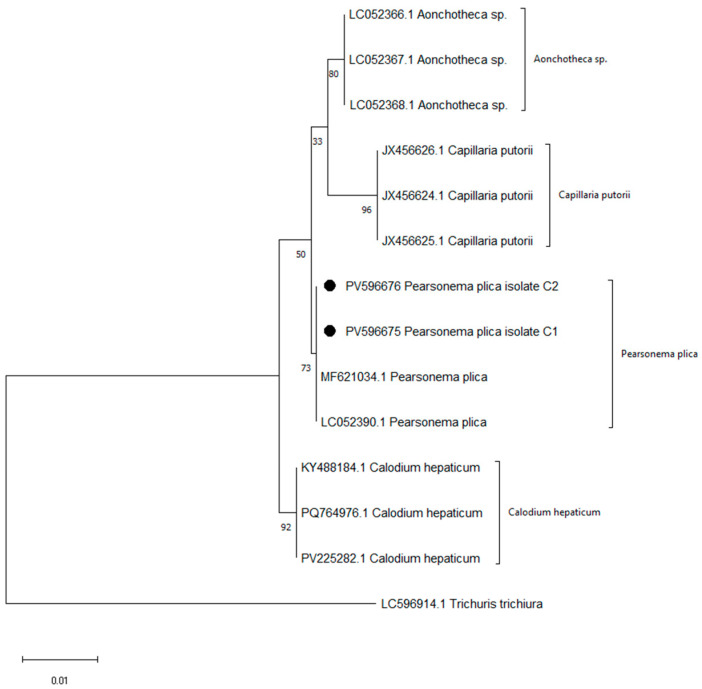
Maximum likelihood phylogenetic tree of *Pearsonema plica* isolates (PV596675, PV596676; indicated by ●) and related Capillariidae species.

A parasitological examination of urine sediment from 88 hunting dogs revealed the presence of *P. plica* eggs (Figure 2), with a prevalence of 20.45% (18 out of 88 dogs). The results from conventional PCR indicated the amplification of a fragment (approximately 620 bp) of the 18S rRNA gene of the urinary nematode, strongly suggesting that the detected parasite in the urine of the dog patients belongs to *P. plica* (Figure S1). BLAST analysis showed 100% sequence similarity with the top 10 hits, all of which were identified as *P. plica*. Phylogenetic analysis confirmed that our two isolates (PV596675/C1 and PV596676/C2) clustered with reference sequences of *P. plica* (MF621034.1 and LC052390.1), thereby validating their identity. In contrast, *Calodium hepaticum*, *Aonchotheca* sp., and *Capillaria putorii* formed distinct and well-supported clades, with *Trichuris trichiura* serving as an outgroup (Figure 3).

Descriptive statistics and multinomial logistic regression results are presented in Table 1. Reference categories are indicated as Ref.val. = 1. Females compared to males (Ref.val. = 1) showed no significant difference in outcome (OR = 1.17, 95% CI: 0.11–13.00, *p* = 0.89). Dogs aged 2–8 years and >8 years, compared to <2 years (Ref.val. = 1), were not significant predictors (OR = 0.53, 95% CI: 0.11–2.55, *p* = 0.43). No significant associations were found for housing conditions or feeding type. Dogs that hunted within the past 2 months had the highest proportion of positive cases (54.54%). Compared to them, dogs that hunted 4 months ago had a lower probability of the outcome, and those that hunted 6 months ago had the lowest (OR = 0.12, 95% CI: 0.02–0.68, *p* < 0.05), indicating that more recent hunting increases risk. Regular deworming was associated with the lowest probability of the outcome (4.00%). Dogs occasionally dewormed had an increased risk (31.82%; OR = 7.96, 95% CI: 1.71–37.11, *p* < 0.001), and those dewormed irregularly had the highest risk (56.25%). Dogs without symptoms had a significantly lower probability of the outcome (7.94%) compared to dogs with symptoms (52.00%; OR = 0.07, 95% CI: 0.010–0.58, *p* < 0.05). No significant associations were observed (*p* > 0.05) in region, season, and urine sampling method.

## 4. Discussion

In this research, we analyzed urine samples from hunting dogs to determine the presence of *P. plica*. The study was conducted across five districts in Serbia. The highest number of positive samples was found in hunting dogs from the Kolubara and Toplica districts. This finding is not surprising, considering that *P. plica* was first diagnosed in Serbia in red foxes (70.6%) within the Kolubara district [4]. The overall prevalence of *P. plica* infection in this study was 20.45%. The relatively high prevalence observed (20.45%) should therefore be interpreted with caution, as it may be influenced by the inclusion of dogs with urinary symptoms and by targeted sampling in areas of known parasite occurrence. In addition, the absence of a structured random sampling design limits population-level inference. Despite these constraints, the findings provide valuable preliminary data and highlight the need for larger, systematic studies to confirm the epidemiological trends suggested here. Data regarding *P. plica* infection in domestic carnivores are quite limited. This scarcity may be attributed to the life cycle of the parasite. Additionally, dogs that have limited contact with the external environment are less likely to be exposed to *P. plica*. However, because hunting dogs are more active and spend time outdoors, their risk of infection is higher. Consequently, existing data indicate that *P. plica* infection is primarily diagnosed in cats, which have more frequent interactions with the external environment.

Canine urinary capillariosis has mainly been reported as isolated cases in several countries, including France [11], the Netherlands [13], Switzerland [12,16], Poland [19], northern and central Italy [15,18,20], western Slovakia [21], Greece [22], and the United States [9,10]. Higher prevalence rates of this endoparasite are primarily observed in wild carnivores, particularly foxes. In our previous research, we found a prevalence of 70.6% in foxes in Serbia [4]. Similar findings have been noted in red fox populations across Europe [1,32,33,34] and in neighboring countries [2,3,20,22,35,36,37]. Urinary capillariosis has also been confirmed in golden jackals, with reported prevalences of 45% in Hungary [38] and 16.4% in Bulgaria [37]. Additionally, it has been identified in 7% of wildcats in certain regions of Germany [14].

PCR amplification and phylogenetic analysis were conducted to confirm the identity of the nematode isolates. The sequences obtained consistently grouped with reference sequences of *P. plica*, which supported the morphological identification and ruled out other closely related species in the Capillariidae family. Although the bootstrap support for the *P. plica* cluster was moderate, the pattern of clustering clearly confirmed the species identity of our isolates.

In most cases, *P. plica* eggs were found in dogs that had been hunting two months prior to sampling. This observation aligns with the known biology of the parasite, as the prepatent period for this nematode is approximately eight weeks. After this period, mature female worms begin to release eggs into the urinary bladder, which are then excreted into the environment through urine [39]. The intense shedding of eggs lasts for about 2.5 months, after which the number of eggs excreted gradually decreases and eventually stops, making it unlikely to detect them in urine samples afterward [40]. Consequently, lower prevalence rates of eggs were noted in dogs that had gone hunting four months or more before urine collection.

Infection with *P. plica* is rarely linked to clinical disease and is often diagnosed incidentally during urine sediment examinations. However, heavily infected animals may exhibit symptoms of clinical cystitis, edema, and muscle membrane hyperplasia. As a result, this infection can lead to issues such as pollakiuria, dysuria, and hematuria [15,41,42]. Our research found that *P. plica* was predominantly present in dogs with urinary symptoms, suggesting that clinically significant, high-intensity infections were more common among the dogs we examined. Komorová et al. [21] reported three isolated clinical cases of *P. plica* infection in dogs diagnosed in western Slovakia. In one clinically healthy dog, the infection was detected incidentally during a urine sediment examination. The other two dogs displayed nonspecific clinical signs, including apathy, loss of appetite, vomiting, polydipsia, and frequent urination. Similarly, Derakhshandeh et al. [43] described a case involving a 1-year-old neutered Shih Tzu Terrier that exhibited symptoms of lethargy, anorexia, ataxia, and pollakiuria. Furthermore, the findings of Studzińska et al. [19] indicated that the clinical course of *P. plica* infection may include blood eosinophilia, hematuria in urine, and the presence of numerous struvite crystals, erythrocytes, leukocytes, and bacteria, often without other symptoms.

In this study, positive samples were primarily found in urine collected through cystocentesis, with a smaller number obtained via spontaneous urination. Cystocentesis is recommended as the preferred sampling method to ensure reliable results, as it helps avoid fecal contamination by eggs of other capillariid species, such as *C. aerophila* and *C. boehmi*. These eggs may be mistaken for those of *P. plica* due to their similar morphology, especially by inexperienced diagnosticians [38,44]. In cases where clinical suspicion remains despite initial negative findings, it is advisable to repeat urine sampling after a few days, as female *P. plica* may intermittently excrete eggs [10].

In hunting dogs, regularly dewormed animals show the lowest prevalence of positive findings for *P. plica*, supporting the implementation of a standardized, seasonally aligned protocol (at least 2–4 times per year), with intensified administration after periods of intensive hunting activity, owner education, and routine urinalysis (sediment examination; repeat testing when indicated). Although the European Scientific Counsel for Companion Animal Parasites [23] recommends monthly anthelmintic administration for hunting dogs, in practice continuity and consistency often depend on owners, so protocols vary among individuals [23]. In this context, combining clear, seasonal deworming plans with targeted owner counseling and laboratory monitoring represents a pragmatic approach that can reduce infection risk.

This study has several limitations that should be taken into account when interpreting the results. The selective urine sampling approach, focused primarily on hunting dogs with or without urinary symptoms, resulted in a relatively small sample size. Consequently, the obtained prevalence may not accurately reflect the true infection rate in the broader hunting dog population. Furthermore, the absence of quantitative urinalysis data limited the ability to correlate parasite load with the severity of clinical signs or other urinary parameters. Future studies with a larger, randomly selected sample and integrated clinical and laboratory data are needed to better clarify the epidemiological and clinical significance of *P. plica* infection in dogs.

## 5. Conclusions

The presence of *P. plica* in hunting dogs indicates that the canine urinary capillariosis remains insufficiently investigated, mainly due to numerous limiting factors. Factors that complicating diagnosis include the long prepatent period, the frequent occurrence of asymptomatic and subclinical infections caused by low parasite burdens, and the intermittent release of small numbers of eggs. Also, the lack of awareness and insufficient knowledge among veterinarians regarding the potential presence of *P. plica* in dogs make timely and accurate diagnosis even more difficult. Considering these challenges, it is reasonable and justified to assume that *P. plica* infection in dogs is present in Serbia, although objective data on its true distribution are still lacking. This report emphasizes the importance of recognizing parasitic infections in domestic carnivores accompanied by urinary tract disorders and highlights the need for further epidemiological studies in this field.

## Figures and Tables

**Table 1:** Multinominal logistic regression of prevalence and risk factors associated with *Pearsonema plica* infection in the examined hunting dogs.

Variable	Category	Ref.val.	N	n	%	β	SE	*p*	OR	95% CI
Sex	Male	1	48	8	16.67	16.67	1.22	1.22	1.17	0.10–13.00
	Female	2	40	10	25.00					
Age	<2 years	1	31	5	16.13	−0.63	0.79	0.43	0.53	0.11–2.55
	2–8 years	2	47	12	25.53					
	>8 years	3	10	1	10.00					
Housing	Kept in yard	1	41	8	19.51	−0.52	0.77	0.50	0.59	0.13–2.7
	Kept in kennel	2	24	5	20.83					
	Kennel with outdoor run	3	23	5	21.74					
Feeding	Mixed	1	35	5	14.29	−0.24	0.85	0.77	0.78	0.15–4.14
	Combined	2	41	12	29.27					
	Commercial	3	12	1	8.33					
Hunting activity	Within past 2 months	1	22	12	54.54	−2.12	0.89	0.05	0.12	0.02–0.68
	Within past 4 months	2	38	6	15.79					
	Within past 6 months	3	28	0	0.00					
Deworming	Regular	1	50	2	4.00	2.07	0.79	0.001	7.96	1.71–37.11
	Occasional	2	22	7	31.82					
	Irregular	3	16	9	56.25					
Urinary symptoms	Present	1	25	13	52.00	−2.57	1.03	0.05	0.07	0.01–0.58
	Absent	2	63	5	7.94					
Region	Branicevo District	1	17	2	11.76	−0.24	0.39	0.53	0.78	0.37–1.67
	South Backa District	2	19	5	26.32					
	Kolubara District	3	21	3	14.29					
	Toplica District	4	20	5	25.00					
	West Backa District	5	11	3	27.27					
Season	Autumn	1	37	6	16.22	−0.01	0.73	0.99	0.99	0.23–4.16
	Winter	2	24	7	29.17					
	Spring	3	27	5	18.52					
Sampling method	Spontaneous urination	1	53	3	5.66	1.89	1.12	0.09	6.67	0.73–60.33
	Cystocentesis	2	35	15	42.86					

N—total number of samples; n—positive samples; β—regression coefficient represents the log odds change for the outcome; SE—standard error of β; OR—odds ratio; 95% CI—confidence interval of OR; Ref.val.—1 indicates the reference category, all other categories are compared to this value.

## Data Availability

The data presented in this study are available on request from the corresponding author.

## Supplementary Materials

The following supporting information can be downloaded at https://www.mdpi.com/article/10.3390/ani15203025/s1: Figure S1: PCR products on 1.5% agarose gel.
