# Supplementary material for: Prevalence and Risk Factors for Pearsonema plica Infection in Hunting Dogs in Serbia
Source: Animals (Basel). 2025 Oct 18;15(20):3025. doi: 10.3390/ani15203025 (PMC12562199; doi:10.3390/ani15203025)
Supplement: Supplementary file 1 [file animals-15-03025-s001.zip › animals-3910006-supplementary.pdf]

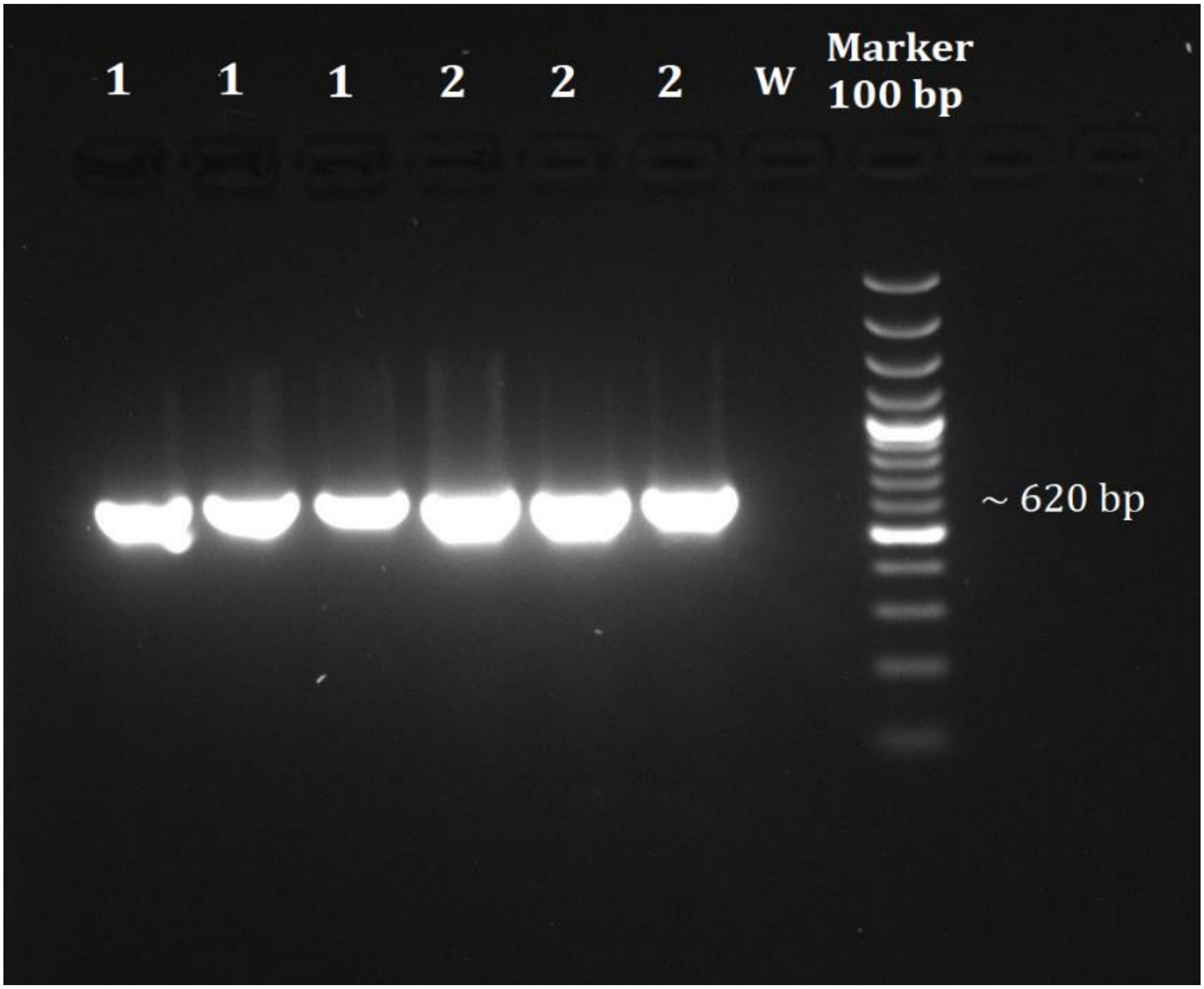

Supplementary Figure S1. PCR products on 1.5% agarose gel. From left to right: samples on positions 1, 2 and 3 (marked No 2) are the first urine sample in triplicate, and samples on positions 4, 5 and 6 (marked No 2) are the second sample in triplicate. W- negative control; and Marker 100 bp. The PCR band of ~ 620 bp presents the part of 18S rRNA gene of urinary Trichuridae nematodes.
